# The Mediating Role of Maladaptive Metacognitive Beliefs between Adverse Childhood Experiences and Trait Anxiety

**DOI:** 10.1192/j.eurpsy.2024.288

**Published:** 2024-08-27

**Authors:** D. Horváth, B. Kovács-Tóth, B. Oláh, Z. Fekete

**Affiliations:** ^1^Department of Behavioural Sciences, University of Debrecen, Debrecen, Hungary

## Abstract

**Introduction:**

Adverse childhood experiences (ACE) have a significant negative impact on health. ACEs lead to more pronounced trait anxiety, among others, which serves as a basis for various mental and somatic symptoms. Recent findings suggest that the fact that individuals with more ACEs also have more maladaptive metacognitive beliefs may contribute to the development of these symptoms.

**Objectives:**

We aim to study the possible mediating role of maladaptive metacognitive beliefs, resulting from adverse childhood experiences, on trait anxiety.

**Methods:**

Data was collected online, anonymously, in a non-clinical population of adults over 18 years of age. The sample consisted of 304 subjects (84.21 % women, 15.79 % men). The applied questionnaires included a demographic questionnaire, the Adverse Childhood Experiences Questionnaire 10 item version, the Meta-Cognitions Questionnaire, and the Spielberger Trait Anxiety Questionnaire. The system of correlations between the examined variables was explored using structural equation modeling (SEM). The study was carried out with ethical approval and in accordance with the Declaration of Helsinki.

**Results:**

Our results confirm that ACEs have a significant impact on all the measured dimensions of maladaptive metacognitive beliefs. The direct effect of ACEs on adult trait anxiety is also significant. The results of the study on indirect effects support the joint mediating role of the five metacognitive dimensions. The strongest significant mediating effect was found for the uncontrollability and dangerousness of negative beliefs about worry. Both the direct and indirect effects of cognitive self-consciousness on adult trait anxiety are negative, which means that the more the cognitive self-consiousness is characteristic of someone, the lower the degree of trait anxiety in adulthood is.

**Conclusions:**

Our results confirm the mediating effect of metacognitive beliefs on trait anxiety in the context of adverse childhood experiences, which points to the importance of further research on metacognition among the population that suffered early adversities. One of the limitations of the study roots in online data collection: the examined sample is not representative. Moreover, to extend the results, it is recommended to repeat the study on a clinical population. This would enable us to compare our results with those of the clinical population, which could provide further important results in the field of metacognition and adverse childhood experiences.

**Disclosure of Interest:**

None Declared

